# The association between serious upper gastrointestinal bleeding and incident bisphosphonate use: a population-based nested cohort study

**DOI:** 10.1186/1471-2318-13-36

**Published:** 2013-04-20

**Authors:** Jennifer A Knopp-Sihota, Greta G Cummings, Joanne Homik, Don Voaklander

**Affiliations:** 1 Faculty of Health Disciplines, Athabasca University, Athabasca, Alberta, Canada; 2 Faculty of Nursing, University of Alberta, Edmonton, Alberta, Canada; 3 Department of Medicine, University of Alberta, Edmonton, Alberta, Canada; 4 Alberta Centre for Injury Control and Research, School of Public Health, University of Alberta, Edmonton, Alberta T6G 2E1, Canada

**Keywords:** Osteoporosis, Bisphosphonates, Adverse events, Upper gastrointestinal bleed, Claims data

## Abstract

**Background:**

Oral bisphosphonates are commonly used to prevent / treat osteoporosis. However, bisphosphonate treatment is not without risk and serious adverse effects, including upper gastrointestinal bleeding (UGIB) have been described. We sought to determine if new users of bisphosphonate drugs were more likely to suffer a serious UGIB within 120 days of drug initiation.

**Methods:**

This was a population-based nested cohort study utilizing administrative healthcare data in British Columbia, Canada. Community based individuals ≥ 65 years with a new prescription for a bisphosphonate between 1991 and 2007 were included. A multivariate logistic regression model was used to examine the relationship between older age and the development of a serious UGIB within 120 days of new exposure to oral bisphosphonate drugs.

**Results:**

Within the exposure cohort (n = 26,223), 117 individuals had suffered a serious UGIB within 120 days of incident bisphosphonate use. Cases tended to be > 80 years old, and were significantly more likely to have had a past history of gastric ulcer disease, a remote history of serious UGIB, and had been dispensed proton pump inhibitor (PPI) medications (p < 0.001 for all comparisons). After adjustment for confounding covariates, those > 80 years were more than twice as likely to suffer a UGIB when compared to those ≤ 80 years (adjusted OR = 2.03; 95% CI 1.40–2.94). A past history of serious UGIB was the strongest predictor of UGIB within 120 days of incident bisphosphonate use (adjusted OR = 2.28; 95% CI = 1.29–4.03) followed by PPI use (adjusted OR = 2.04; 95% CI = 1.35–3.07). Males were 70% more likely to suffer an UGIB compared to females (adjusted OR = 1.69; 95% CI = 1.05–2.72).

**Conclusions:**

Upper GIB is a rare, but serious, side effect of bisphosphonate therapy more often afflicting older individuals. At the same time, concern about potential rare adverse events should not discourage clinicians from prescribing bisphosphonate drugs, particularly in older patients who have already sustained a fragility fracture. Clinicians must remain cognizant of potential adverse events associated with bisphosphonate use and should routinely ask about pre-existing GI disorders and concurrent medication history prior to prescribing these drugs.

## Background

The routine management of osteoporosis should target all aspects of the disease, including maximizing and preserving bone mass and preventing future fractures through pharmacotherapy and lifestyle modification [1]. The mainstay of osteoporosis treatment includes calcium and vitamin D, along with an antiresorptive agent (usually a bisphosphonate drug) [1,2]. In addition to osteoporosis treatment, bisphosphonate drugs are also used for less common conditions such as Paget’s disease of the bone and malignancy related bone pain and bone loss [3]. Bisphosphonates have been shown to rapidly reduce bone-remodeling, thus increasing bone mass density, and are associated with the largest reduction in fracture risk when compared to other therapies [4].

However, bisphosphonate treatment is not without risk and serious adverse drug reactions (ADRs), including osteonecrosis of the jaw [5], oral and gastric carcinomas [6-8], atypical femur fractures [9,10], and upper gastrointestinal bleeding (UGIB), although infrequent, have been described but remain controversial [11-15]. Drug induced dysphagia, esophagitis, and gastric ulcers are the most common of the gastrointestinal (GI) ADRs associated with oral bisphosphonate therapy. Upper GI effects are largely linked to improper drug administration regimes (i.e. insufficient water intake and failing to sit upright following medication ingestion) [15] and the local effects of bisphosphonates on the esophagus or gastric mucosa. In fact, ADRs, defined as any injury resulting from medication use that occurs due to pharmacologic properties of the drug [16], are thought to be the reason for bisphosphonate discontinuation in up to 20% of subjects [17].

Although several observational studies have reported on minor GI adverse effects such as nausea, dyspepsia, and epigastric pain, few large population based studies have assessed the more serious adverse events of drug-induced acute UGIB. One large population based case–control study utilized a Canadian population to compare the risk of UGIB between users of bisphosphonates alone and users of bisphosphonates and NSAIDS concurrently [18]. This study by Etminan et al. utilized a previously established community-based cohort of individuals who had undergone a prior coronary revascularization procedure; of note, this population was highly selected, and therefore may not necessarily be representative of the general population. They found no evidence of an increase in the risk of UGIB among current users of bisphosphonate, but did find a two fold increase in risk for concurrent users of bisphosphonates and NSAIDs. Another population based study investigated the excess risk of hospitalizations for UGIB associated with alendronate use. These authors conducted a matched case control study and found a higher unadjusted rate of UGIB for bisphosphonate users; after controlling for confounding variables such as prior osteoporosis fractures, they found no significant differences in risk between cases and controls [19].

A different study, based on a Danish population, investigated the risk of esophageal and gastric events in a group of older adults [20]. For their endpoint, these authors did not distinguish between those developing minor GI conditions such as esophagitis with those suffering the more serious event of a UGIB. They measured the rates of adverse GI events both before and after the initiation of various osteoporosis medications. The authors found no difference as they discovered that GI event rates were increased both before and after initiation of the drugs. Other studies have focused primarily on either daily versus weekly bisphosphonate dosing (finding fewer GI events with weekly dosing) or have compared the GI tolerability of two separate bisphosphonate medications. For example, one such study by Cadarette et al. compared the GI safety between weekly doses alendronate and risedronate. These authors found no important differences between the two weekly preparations [21].

### Objectives

Definitive evidence of a causal relationship between bisphosphonate therapy and serious ADRs, particularly UGIBs is lacking. Furthermore, there remain concerns about the risks of long term treatment particularly among older patients with increased co-morbidity. For that reason, we sought to examine the risk of serious UGIB among incident oral bisphosphonate users in British Columbia (BC), Canada. Specifically, we sought to determine if community dwelling older adults (> 80 years), who were new users of bisphosphonate drugs (incident users), were more likely to suffer a serious UGIB within 120 days of drug initiation in comparison to younger (≤ 80 years) incident users of the same therapy.

## Methods

### Study design and data source

We performed a population-based retrospective nested cohort analysis utilizing de-identified administrative healthcare data derived from the British Columbia Linked Health Database (BCLHD). This database contained comprehensive healthcare utilization data for nearly all residents of BC, Canada (population 4.1 million, 2006 Statistics Canada census data). The BCLHD, which integrates health service records, population health data, and census statistics, makes it possible to link administrative records anonymously at the individual level by using a unique personal health number (PHN). The BCLHD has been used in numerous healthcare and health services research projects since 1996; thus, this database is well suited to explore clinical questions. Prior to accessing data, ethics approval was received from the Health Research Ethics Board at the University of Alberta.

### Identification of the study sample

We used a previously assembled cohort of all persons aged ≥ 65 years who had suffered a fracture between April 1, 1991 and March 31, 2002 (n = 81,870). Within the fracture group, 63% had sustained a hip fracture, 17% an arm / wrist fracture, 9% a pelvic fracture, 7% a vertebral fracture, and 4% had sustained a rib fracture. In addition, a comparison group consisting of a random sample (n = 142,077) of non-fracture subjects registered in the BCLHD over the same time period were added to the cohort for a total source cohort of 223,947 individual patients. Follow-up of this population continued an additional 5 years until March 31, 2007. As we focused only on those prescribed bisphosphonates, the fracture cohort was not separated from the comparison group nor was it delineated in the analysis. The study group all had continuous enrolment in the PharmaCare prescription benefits plan (Fair PharmaCare or Plan B) during the study index period. PharmaCare is BC’s public drug insurance program that assists residents in paying for eligible prescription drugs and certain medical supplies [22]. The PharmaCare dataset includes patient level prescription drug expenditures for community dwelling individuals and residents of licensed residential care facilities. Between 1991 and 2007 the population aged ≥ 65 years in the province grew from approximately 428,088 to 616,804 [23].

From this initial cohort, we constructed a bisphosphonate “exposure” cohort using PharmaCare data to identify all incident users of bisphosphonate drugs; incident users were defined as patients ≥ 65 years who had been dispensed an oral bisphosphonate drug during the study index period (n = 26,518 [11.8%]). As we were primarily interested in ADRs associated with new users of the drug, we excluded any person who had received an oral bisphosphonate within the previous 365 days of the index date (i.e. bisphosphonate wash-out period). Oral bisphosphonate drugs available during the index period and covered by the PharmaCare plan included alendronate (Fosamax), etidronate (Didrocal), and risedronate (Actonel). Although there are currently other manufacturers and brand names for alendronate (the patent on aledronate expired in 2008), Fosamax was the only formulation available during the study index period as the only manufacturers were the patent holders.

The index date was defined as the date of first claim for an oral bisphosphonate prescription during the index period. Gastrointestinal symptoms appear in similar rates, regardless of the specific oral bisphosphonate or dosing regimen; therefore, we included all available bisphosphonate formulations regardless of whether the dosing was daily or weekly [21,24].

### Identification of cases

To identify nested cases (those sustaining an UGIB within 120 days of incident bisphosphonate use) within the bisphosphonate exposure cohort, we then linked to the Discharge Abstract Database (DAD) to identify patients with a diagnosis of UGIB. The DAD contains demographic, administrative, and clinical data for hospital discharges (inpatient acute, chronic, and rehabilitation) and day surgeries [25]. The data are collected per patient admission and contain up to 16 diagnosis codes. We included patients ≥ 65 years with a hospital admission (primary, secondary, or other) for an acute UGIB occurring within 120 days from the dispensation of an oral bisphosphonate prescription (n = 412). We defined a serious UGIB as those patients who had been admitted to hospital with an acute and / or unspecified hemorrhage and / or perforation of either a gastric, duodenal, peptic, or gastrojejunal ulcer or an unspecified hemorrhage of the gastrointestinal tract (See Additional file 1 for the specific ICD-9 codes used). Rates of GI related adverse events tend to be the highest in the first few months following the initiation of the therapy; thus, a 120 day time period was deemed sufficient to capture the majority of adverse events [26-29]. Oral bisphosphonates are dispensed in 100-day quantities under the PharmaCare plan; therefore, patients will be considered at risk for 120 days after a dispensed prescription. This time period allows for the consumption of the prescription, subsequent early refills (i.e. not beginning the prescription immediately upon filling because the previous prescription of a medication other than a bisphosphonate was not complete), and possible non-adherence (i.e. prescription lasting longer than intended). Other researchers have used this approach to analyze rare events associated with prescription drug use with administrative data [21,30].

**Figure 1:**
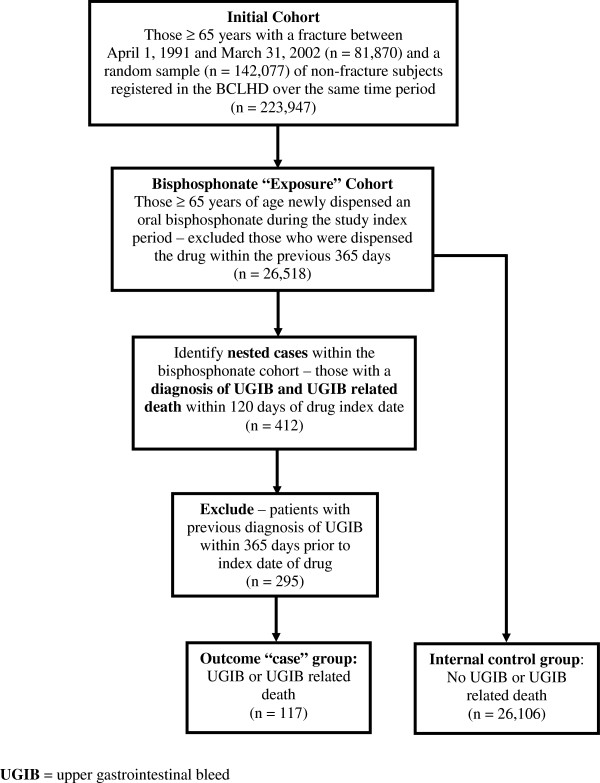
**Identification of the study sample.** UGIB = upper gastrointestinal bleed.

To identify additional cases of UGIB related deaths (without hospitalization), we then linked to the BC Vital Statistics death events registry (for cause of death). This registry includes all deaths that occur within the province [31]. Patients with a previous diagnosis of UGIB, requiring hospitalization or related death, within 365 days prior to the index date of drug dispensation were excluded (n = 295) leaving 26,223 individual patients in the exposure cohort of which 117 had sustained an UGIB. As this was a prevalent controls design, those who had been dispensed oral bisphosphonate therapy, but did not suffer a UGIB or related death by the end of the study period, acted as the internal control group (n = 26,106). See Figure 1 for sample selection procedures.

### Outcome measures and analysis

All analyses were stratified by UGIB status and age and descriptive statistics were used to summarize the characteristics of the population. We planned to firstly explore relationships between the dependent variable (UGIB within 120 days of incident oral bisphosphonate use), age group (the main independent variable), and the covariates of sex, co-morbidity, any past history of gastric ulcer disease, past history of serious UGIB requiring hospitalization, and concurrent use of prescription nonsteroidal anti-inflammatory drugs (NSAIDs), antiplatelet / anticoagulation medications, and the use of proton pump inhibitors (PPIs) using Pearson’s chi square (χ^2^) statistics, with alpha (p) set at 0.05 to determine the statistically significant differences between groups. Where there were unexpected findings, we planned to conduct a post hoc analysis to further explore the relationships between variables. As this was a population-based study, a sample size calculation was not warranted.

To compare the incidence rates (IR) for UGIB between age groups we calculated the person-time of exposure based on the assumption that the exposure cohort were all at risk (exposed) for 120 days post incident bisphosphonate use. To compare the relative risk (RR) for UGIB among incident bisphosphonate users (in the immediate 120 day time period) with the general population, we used a population rate for UGIB as 1 per 1,000 person-years [32,33].

Using univariate logistic regression techniques, we initially assessed the unadjusted odds ratios (OR) and 95% confidence intervals (CI) between the dependent variable (UGIB within 120 days of incident oral bisphosphonate use), age group (the main independent variable), and all covariates. Using the techniques described by Hosmer and Lemeshow, we planned to include only those variables that were statistically significant (p < 0.20) in the multivariate analysis [34]. The co-morbidity (p = 0.57 to 0.49) and anticoagulation (p = 0.85) variables were not statistically significant; however, we considered both of these variables to be clinically important so we included them in the initial multivariate model (both were later removed from the model as they continued to show no statistical significance). See Table 1 for the unadjusted, univariate regression results. For the multivariate analysis, we used the *Enter* procedure in which all independent variables are entered in a single step and then tested for the possibility of statistical interaction between the main independent variables (age group) and all other covariates. We pre-specified that we would consider only interaction terms that achieved a level of statistical significance of p < 0.10 [34].

**Table 1:** **Characteristics of the bisphosphonate exposure cohort stratified by upper gastrointestinal bleeding status **^**a**^

	**UGIB**		**Unadjusted odds ratio (95% CI)**	**p -Value**
	**n = 117 (0.4)**			
	**≤ 80 years n = 47 (40.2)**	**> 80 years n = 70 (59.8)**		
**Age (y)**				
≤ 80	47	-	(reference)	
> 80	-	70	2.08 (1.44, 3.01)	0.000
**Sex **^**c**^				
Female	39 (83.0)	57 (81.4)	(reference)	
Male	8 (17.0)	13 (18.6)	1.72 (1.07, 2.76)	0.025
**Comorbid conditions **^**c**^				
None	2 (4.3)	4 (5.7)	(reference)	
1 – 3	9 (19.1)	17 (24.3)	1.29 (0.51, 3.15)	0.571
4 - 6	28 (59.6)	38 (54.3)	1.61 (0.70, 3.71)	0.267
7 - 15	8 (17.0)	11 (15.7)	1.69 (0.55, 3.48)	0.487
**Past history of gastric ulcer disease **^**b**^				
No	35 (74.5)	58 (82.9)	(reference)	
Yes	12 (25.5)	12 (17.1)	2.21 (1.41, 3.46)	0.001
**Past history of serious GI bleeding **^**b**^				
No	41 (87.2)	62 (88.6)	(reference)	
Yes	6 (12.8)	8 (11.4)	2.79 (1.59, 4.90)	0.000
**NSAIDs (oral) **^**d**^				
No concurrent use	42 (89.4)	65 (92.9)	(reference)	
Concurrent use	5 (10.6)	5 (7.1)	0.42 (0.22, 0.80)	0.009
**Anti-platelet / anti- coagulant drugs (oral) **^**c**^				
No concurrent use	40 (85.1)	64 (91.4)	(reference)	
Concurrent use	7 (14.9)	6 (8.6)	0.85 (0.48, 1.51)	0.848
**Proton pump inhibitor drugs (oral) **^**b**^				
No concurrent use	33 (70.2)	50 (71.4)	(reference)	
Concurrent use	14 (29.8)	20 (28.6)	2.03 (1.36, 3.03)	0.001

^**a**^ Data are shown as number (percentage) unless otherwise indicated.

^**b**^ p < 0.001.

^**c**^ Not significant.

^**d**^ p < 0.05.

**CI** = confidence interval; **GI** = gastrointestinal; NSAID = **UGIB** = upper gastrointestinal bleed.

We then checked for confounding of the variables that were removed from the model; as none of the beta coefficients changed by more than 15% we were satisfied that neither of the variables were statistically significant predictors of the dependent outcome nor confounders. There were no statistically significant interactions between the main independent variable (age group) and the remaining variables; therefore, none of the interaction terms were retained in the final model. The final multivariate model included the variables: age, sex, past history of gastric ulcer disease, past history of serious UGIB, concurrent use of prescription NSAIDs and the use of PPIs. The adjusted ORs were considered statistically significant if the 95% CI did not include 1. All analyses were conducted using SPSS version 19.0 (IBM SPSS Statistics).

### Explanatory variables: covariates

Based on clinical relevance and known UGIB risk factors identified in previous research [32,35-38], we planned to control for the following potential confounders: age (grouped as ≤ 80 years and > 80 years), sex (female, male), remote past history (> 1 year prior) of serious UGIB requiring hospital stay (ICD-9 codes 530–535 or 578 in the DAD file), past history of gastric ulcer disease (ICD-9 code of 530–534 in the Medical Services Plan [MSP] or DAD file), concurrent use of prescription oral NSAIDs, and oral antiplatelet / anticoagulation medications. Concurrent use of a PPI was also examined as other researchers have used the use of PPIs as a marker or proxy for the presence of preexisting GI disease. See Additional file 2 for the specific medications we included in the analysis.

To assess the prevalence of co-morbidity, *a priori* we assembled a constellation of 15 chronic disease diagnoses, which are often symptomatic and are thus important in predicting morbidity and mortality. Co-morbidity is defined as the co-existence of two or more chronic conditions or impairments that have an impact upon patient independence and survival [39]. Specifically, the co-morbidities included in the analysis were: cancer, cerebrovascular disease, diabetes, cardiovascular disease, hypertension, osteoporosis, osteoarthritis, rheumatoid arthritis, neurotic disorders, depression / psychosis, incontinence, Parkinson’s disease, chronic obstructive pulmonary disease, and asthma. The diagnoses chosen were largely based on those identified in other co-morbidity indices (i.e. The Charlson Index [40] and Elixhauser’s method [41]) and have been used in previous studies [42]. Co-morbidity was identified by searching the MSP payment file to identify those patients with at least two primary care visits for the same diagnosis within the last two years of the study period. The MSP is the province’s universal health insurance program, and contains data on outpatient services provided by fee for service practitioners. One diagnosis code is reported per patient encounter; this code is assumed to be the primary diagnosis or reason for the visit or service [43]. In order to compare co-morbidity between subjects, we simply added the number of diagnoses. This “disease counting” approach is less complex and studies have shown them to be as effective (if not more effective) as other more complex measurements in predicting and controlling for co-morbidity such as the Charlson Index [44-47].

## Results

### Study population

After exclusion criteria were applied, we identified 26,223 individual patients in the exposure cohort. The mean age of the sample was 78.8 years (SD 6.9; range 65–104 years), and 88% of the subjects were female. The majority of the cohort had between 4 and 6 co-morbid conditions (51%), 10% had a past medical history of gastric ulcer disease, 5% had a remote history of serious UGIB, 18% used NSAIDs, 13% used antiplatelet / anticoagulant prescriptions drugs, and 17% used PPIs.

Within the exposure cohort, 117 (0.4%) individual patients had suffered a serious UGIB (116 requiring hospitalization and one death) within 120 days of incident bisphosphonate use; the remaining 26,106, acted as the internal control group. Those who developed an UGIB (the 117 cases) tended to be > 80 years old (60%), and when compared to those who did not suffer a UGIB, they were significantly more likely to have had a past history of gastric ulcer disease (21% vs. 11%), a remote history of serious UGIB (12% vs. 5%), and had been dispensed PPI medications (29% vs. 17%) (all were p < 0.001). Cases were less likely to have been dispensed NSAIDs compared to controls (9% vs. 18%; p < 0.05). There were no statistical differences in sex, level of co-morbidity, or use of antiplatelet / anticoagulant prescriptions drugs between groups. See Table 2 for characteristics of the exposure cohort stratified by UGIB status.

**Table 2:** **Characteristics of bisphosphonate exposure cohort stratified by upper gastrointestinal bleeding status **^**a**^

	**UGIB**	***No *UGIB**	**Total**
	**n = 117 (0.4)**	**n = 26 106 (99.6)**	**N = 26 223**
**Age (y) **^**b**^			
≤ 80	47 (40.2)	15,213 (58.3)	15,260 (58.2)
> 80	70 (59.8)	10,893 (41.7)	10,963 (41.8)
**mean ± SD**	81.8 ± 6.9	78.8 ± 6.9	78.8 ± 6.9
**Sex **^**c**^			
Female	96 (82.1)	23,030 (88.2)	23,126 (88.2)
Male	21 (17.9)	2,934 (11.2)	2,955 (11.3)
Unknown	0	142 (0.5)	142 (0.5)
**Comorbid conditions **^**c**^			
None	6 (5.1)	1,936 (7.4)	1,942 (7.4)
1 – 3	26 (22.2)	6,490 (24.9)	6,516 (24.8)
4 - 6	66 (56.4)	13,256 (50.8)	13,322 (50.8)
7 - 15	19 (16.2)	4,424 (16.9)	4,443 (16.9)
**mean ± SD**	4.77 ± 2.1	4.41 ± 2.2	4.41 ± 2.2
**Comorbid conditions (by diagnosis)**			
Cancer ^**c**^	44 (37.6)	9,056 (34.7)	9,100 (34.7)
Cerebrovascular disease ^**c**^	30 (25.6)	5,324 (20.4)	5,354 (20.4)
Diabetes ^**c**^	16 (13.7)	4,269 (16.4)	4,285 (16.3)
Cardiovascular disease ^**b**^	91 (77.8)	16,542 (63.4)	16,633 (63.4)
Hypertension ^**c**^	84 (71.8)	17,437 (66.8)	17,521 (66.8)
Osteoporosis ^**c**^	27 (23.1)	7,354 (28.2)	7,381 (28.1)
Osteoarthritis ^**c**^	66 (56.4)	14,954 (57.3)	15,020 (57.3)
Rheumatoid arthritis ^**c**^	11 (9.4)	3,481 (13.3)	3,492 (13.3)
Neurotic disorder ^**c**^	52 (44.4)	9,941 (38.1)	9,993 (38.1)
Depression / psychosis ^**c**^	60 (51.3)	12,403 (47.5)	12,463 (47.5)
Dementia ^**c**^	27 (23.1)	5,050 (19.3)	5,077 (19.4)
Incontinence ^**c**^	48 (41.0)	9,473 (36.3)	9,521 (36.3)
Parkinson’s ^**c**^	2 (1.7)	835 (3.2)	837 (3.2)
COPD ^**c**^	38 (32.5)	7,910 (30.3)	7,948 (30.3)
Asthma ^**c**^	16 (13.7)	3,533 (13.5)	3,549 (13.5)
**Past history of gastric ulcer disease **^**b**^			
No	93 (79.5)	23,374 (89.5)	23,467 (89.5)
Yes	24 (20.5)	2,732 (10.5)	2,756 (10.5)
**Past history of serious GI bleeding** **(requiring hospital stay) **^**b**^			
No	103 (88.0)	24,895 (95.4)	24,998 (95.3)
Yes	14 (12.0)	1,211 (4.6)	1,225 (4.7)
**NSAIDs (oral) **^**d**^			
No concurrent use	107 ( 91.5)	21,342 (81.8)	21,449 (81.8)
Concurrent use	10 (8.5)	4,764 (18.2)	4,774 (18.2)
**Anti-platelet / anti-coagulant** **prescription drugs (oral) **^**c**^			
No concurrent use	104 (88.9)	22,751 (87.1)	22,855 (87.2)
Concurrent use	13 (11.1)	3,355 (12.9)	3,368 (12.8)
**Proton pump inhibitors **^**b**^			
No concurrent use	83 (70.9)	21,725 (83.2)	21,808 (83.2)
Concurrent use	34 (29.1)	4,381 (16.8)	4,415 (16.8)

^**a**^ Data are shown as number (percentage) unless otherwise indicated.

^**b**^ p < 0.001.

^**c**^ Not significant.

^**d**^ p < 0.05.

**GI** = gastrointestinal; **NSAID** = Non-steroidal anti-inflammatory drug; **UGIB** = upper gastrointestinal bleed.

We explored a number of post hoc relationships between variables of interest. Those with greater co-morbidity were significantly more likely to have been dispensed a NSAID and there was an inverse relationship between NSAID use and the past history of gastric ulcer disease. There were significant positive relationships between PPI use and a past history of gastric ulcer disease and between PPI use and a past history of a serious UGIB.

### Incidence of UGIB

Assuming those in the bisphosphonate exposure cohort were at risk for 120 days, we calculated an overall RR of approximately 14 per 1,000 person-years for UGIB within 120 days of incident bisphosphonate prescription. When stratified by age, those patients > 80 years (RR = 19) had more than two times the incidence of UGIB compared to those ≤ 80 years (RR = 9). The corresponding absolute risk (AR) increase was 13 per 1,000 per years. See Table 3 for crude IRs.

**Table 3:** Crude incidence rates of upper gastrointestinal bleeding within 120 days of incident bisphosphonate drug prescription

	**≤ 80 years**	**> 80 years**	**Total**
Number of UGIB events	47	70	117
Person-time at risk (y)	5,017	3,604	8,621
Incidence rate ^**a**^	9.4	19.4	13.6

^**a**^ Incidence rate (per 1 000 person-years).

**UGIB** = upper gastrointestinal bleed.

### Factors associated with UGIB: logistic regression analysis

A past history of serious UGIB was the strongest predictor of UGIB within 120 days of incident bisphosphonate use (adjusted OR = 2.28; 95% CI = 1.29–4.03). The next strongest predictor of UGIB was the concurrent use of PPI medications. In comparison to those who were not concurrently dispensed a PPI medication, patients with a PPI were significantly more likely to suffer an UGIB within 120 days post initiation bisphosphonate drug (adjusted OR = 2.04; 95% CI = 1.35–3.07). Other statistically significant predictors of UGIB included age > 80 years (adjusted OR = 2.03; 95% CI = 1.40–2.94), a past history of gastric ulcer disease (adjusted OR = 1.90; 95% CI = 1.21–3.01), and male sex (adjusted OR = 1.69; 95% CI = 1.05–2.72). The use of prescription NSAIDs was found to be a significant negative predictor of UGIB (adjusted OR = 0.41; 95% CI = 0.21–0.80). See Table 1 for the unadjusted ORs and Table 4 for factors (the adjusted ORs) predicting UGIB within 120 days of incident bisphosphonate use.

**Table 4:** Logistic regression model: factors predicting gastrointestinal bleeding within 120 days of incident bisphosphonate use

**Variable**	**Adjusted odds ratio (95% CI)**	**p -Value**
**Constant**	0.002	0.000
**Age (y)**		
≤ 80	1 (reference)	
> 80	2.03 (1.40, 2.94)	0.000
**Sex**		
Female	1 (reference)	
Male	1.69 (1.05, 2.72)	0.030
**Past history of gastric ulcer disease**		
No	1 (reference)	
Yes	1.90 (1.21, 3.01)	0.006
**Past history of serious GI bleeding**		
No	1 (reference)	
Yes	2.28 (1.29, 4.03)	0.005
**NSAIDs (oral)**		
No concurrent use	1 (reference)	
Concurrent use	0.41 (0.21, 0.80)	0.008
**Proton pump inhibitor drugs (oral)**		
No concurrent use	1 (reference)	
Concurrent use	2.04 (1.35, 3.07)	0.001

**CI** = confidence interval; **GI** = gastrointestinal; **NSAID** = Non-steroidal anti-inflammatory drug; **UGIB** = upper gastrointestinal bleed.

## Discussion

For many older adults with a diagnosis of osteoporosis, oral bisphosphonate drugs are the first line of treatment. While these drugs are typically safe, there have been reports of serious adverse events. In this population-based nested cohort study, we identified subjects in a Canadian province with universal health care coverage, who were newly dispensed a bisphosphonate drug. Although we found a relatively low rate of UGIBs overall, only 0.4% of the exposure cohort developing this rare event, older patients (> 80 years) were significantly more often affected.

Using logistic regression techniques, we found that older age was an independent risk factor for developing an UGIB within 120 days post bisphosphonate treatment; this relationship remained after controlling for sex, history of gastric ulcer disease, history of serious UGIB, NSAID, and PPI use. Regardless of age, patients who were male, had a past history of gastric ulcer, a more remote UGIB history, and used a PPI, were more likely to suffer a UGIB post bisphosphonate use. Interestingly, patients who had been dispensed NSAIDs concurrently were less likely to develop a UGIB within 120 days of bisphosphonate use.

### Age, sex, and risk of UGIB

The incidence of UGIB (within 120 days of incident bisphosphonate use) was much higher for older subjects; in fact, patients > 80 years of age developed an UGIB two times more often when compared to those ≤ 80 years. This finding is congruent with UGIB (from any cause) in the general population where advanced age has consistently been identified as a risk factor for the increased incidence of UGIB and related mortality [38]. While we recognize that older adults have multiple factors influencing their risk of GI bleeding, UGIB is thought primarily to be related to increased co-morbidity and the use of multiple medications in the older age group [37]. Although we were unable to control for total medication counts per patient in our study, we postulate that the increased co-morbidity in the older group corresponded to an increase in medication use which potentially contributed to greater UGIB risk. Additional factors identified by other researchers include inactivity and disability, both of which have been associated with a higher risk of GI bleeding in older adults [37,48]. Others have attributed the increased incidence to the increased use of NSAIDS in older adults, who are at greater risk of GI toxicity from these agents, as well as a higher prevalence of *Helicobacter pylori* and gastroesophageal reflux disease [49,50]. Peptic ulcer disease is not only strongly associated with *Helicobacter pylori* infection and more common in older adults but is also well known as the most common cause of UGIB [38]. We found that gastric ulcer disease was a strong independent predictor of UGIB; specifically, those with a past history of gastric ulcer disease were 90% more likely to develop an UGIB (within 120 days of incident bisphosphonate use) than those with no history of the disease.

In our study, we were concerned primarily with bisphosphonate medications and their association with UGIB in an older population. Esophagitis and gastropathy, described as mucosal injury to the esophagus and stomach respectively, can be caused by drugs such as bisphosphonates that are known to cause local effects on both areas of the GI tract [15]. Although overt UGIB is an uncommon manifestation of esophagitis or gastropathy, these lesions are implicated as bleeding sources more frequently in the older adult, especially those 80 years and older [51].

We found that regardless of age, males had an approximately 70% increased risk of hospitalization for UGIB than females. This is consistent with previous studies, who have found approximately a two-fold increase in the incidence of UGIB, from any cause, in males than in females; however, mortality rates are similar in both sexes [32,52]. In our study this was not unexpected as the male group was also found to have significantly more co-morbid disease, notably cardiovascular disease, and were more likely to have a past medical history of gastric ulcer disease both of which are associated with an increased risk of UGIB.

### NSAIDs, PPIs, and risk of UGIB

Many studies, of various designs, have focused on NSAID use in relation to UGIBs; for the most part, findings over the last 15 years suggest that current users of NSAIDS have at least a 3 to 5 fold increased risk of UGIB [53]. Contrary to these results, in our study we found that a prescription for a NSAID was the strongest negative predictor of UGIB; those who had been dispensed NSAIDs were two and a half times less likely to suffer a UGIB within 120 days of incident bisphosphonate use. Because of the known GI side effects associated with NSAID use, we initially speculated that perhaps patients, who were prescribed a NSAID, were healthier patients with fewer co-morbid conditions, thus less likely to develop a UGIB. We found that those with greater co-morbidities were significantly more likely to be dispensed a NSAID drug; therefore, the notion that those prescribed a NSAID were healthier did not stand. We did find an inverse relationship between NSAID use and past history of gastric ulcer disease; those with a gastric ulcer were less likely to have been dispensed a NSAID. For that reason, we postulated that the “protective” effect of NSAIDs may stem from their use in patients who do not have a history of gastric ulcer thus making users of NSAIDs perhaps less likely to develop a UGIB. The clinical significance of this finding, however, is unclear and caution in the interpretation of this finding is thus warranted.

We also found that those patients who had been dispensed a PPI medication were two times as likely to develop a UGIB post incident bisphosphonate use. As we used the prescription of a PPI as a proxy for the presence of GI disease, this finding was expected. Those who were at an increased risk, or who have already developed GI disease, are often prescribed drug therapy with either a histamine-2 (H2)–receptor antagonist (typically available as over the counter medications) or a PPI in order to provide mucosal protection [54]. In other words, those patients using a bisphosphonate and a PPI likely had a greater underlying risk of an adverse GI event compared to those persons using a bisphosphonate alone; this would account for the observed increase in UGIB risk for concurrent users of the drugs [19]. This was confirmed during post hoc analysis where we found significant positive relationship between PPI use and both a past history of gastric ulcer disease and past serious UGIB.

### Comparison to previous research

Although a number of randomized controlled trials (RCTs) have reported higher rates of upper GI tract minor irritations in treatment groups (although not reaching statistical significance), there have been no reports of more serious upper GI tract adverse events such as UGIB [55-57]. RCTs typically follow a smaller group of highly selected individuals for a relatively short period of time and are designed primarily to investigate the fracture prevention efficacy of bisphosphonates. Unfortunately, there are few previous population based studies investigating the risk of UGIB associated with bisphosphonate drugs to compare our study results to [18-21].

### Limitations

This study, as with other studies based solely on administrative data, has some limitations that must be recognized. First, bisphosphonates are not only prescribed for the prevention and treatment of osteoporosis, but also for the treatment of certain malignancies and related malignancy complications as well as other serious conditions such as Paget’s disease of the bone. As we exclusively examined oral bisphosphonate preparations, we can be fairly certain that these drugs were not used to treat a malignancy or related complication such as hypercalcemia as these conditions are primarily treated with intravenous bisphosphonate infusions. Furthermore, a diagnosis of Paget’s disease is relatively rare (in comparison to a diagnosis of osteoporosis); moreover, we were interested in adverse events associated with new bisphosphonate use and as we controlled for co-morbidity, we did not think the primary therapeutic use of the drug would have changed the results of our study.

Second, the exposure cohort was compiled based on dispensation of bisphosphonate drugs and may not be an accurate reflection of actual drug consumption rates; therefore, we may have overestimated the use of bisphosphonates and perhaps have then underestimated UGIBs related to the new use of the drug (non-differential misclassification). This type of misclassification of exposure would bias the effect measure toward an apparent null effect. Third, due to the claims-based nature of the dataset, information related to other potentially confounding variables could not be assessed or controlled for. For example, we lacked data on other factors known to be related to UGIBs such as smoking and alcohol consumption, the use of over the counter medications such as NSAIDs (i.e. ibuprofen) and ASA, and the presence of un-diagnosed helicobacter pylori infections [32,58]. Although the inclusion of these variables may have provided a more inclusive description of potential UGIB predictors, we do not believe that controlling for these variables would have altered the results of this study.

Thirdly, we based the outcome diagnosis of UGIB exclusively on discharge abstract coding and have no way of knowing if endoscopy was done and if done, where in fact the GI bleeding originated from (esophagus vs. stomach). We attempted to overcome this limitation by only including those patients who were diagnosed with a “serious” bleed; therefore, we are confident that diagnoses were confirmed in the clinical setting by endoscopy.

Lastly, we would be remiss if we did not acknowledge that the relationship between bisphosphonates and UGIBs may also be complicated by the known fact of an already increased prevalence of GI tract symptoms among older adults [59]. In our study, we did not find differences in co-morbidity between those who developed a UGIB and those who did not. We did find that those who developed a UGIB were more likely to both have a concurrent past diagnosis of gastric ulcer disease as well as a remote history of UGIB. Taken together, this may have accounted for some of the decrease in observed risk in the group who did not develop a UGIB - even after controlling for these variables as confounders.

The strengths of this study include its population-based design, a large sample size, and its use of detailed data made possible by the use of a comprehensive linkable dataset including clinical and prescription drug information of most residents in the province. To our knowledge, this is one of the first comprehensive, population based studies of UGIB incidence associated with new oral bisphosphonate use. We used a strict case definition of UGIB, which has been previously validated; this strategy for identifying cases of UGIB (a rare event) was broad, reducing the likelihood that cases were missed [60]. In addition, our study used a co-morbidity index that had been previously validated with the population and used sophisticated statistical analysis to control for potentially confounding variables. Given the longitudinal nature of the data, it was also possible to examine drug use as both an exposure (bisphosphonate) and an end-point (UGIB) thereby having the advantage of enough accumulated person-time of bisphosphonate exposure for detection of more rare GI associated adverse events. Taken together, these factors ought to contribute to a reliable estimate of the ORs for UGIBs within 120 days of incident bisphosphonate use.

## Conclusion

Upper GIB is a rare, but serious, side effect of bisphosphonate therapy with older patients being affected more often than younger ones. In our study, those incident users of oral bisphosphonate drugs aged > 80 years had a two-fold increase in serious UGIB within 120 days of new drug use. Unfortunately, osteoporosis related fracture risk also increases substantially with age and those older adults are at a much higher risk of morbidity and mortality related to fractures. Concern about potential rare adverse events should not discourage clinicians from prescribing bisphosphonate drugs when there is a high risk of fracture, particularly in older patients many of who have already sustained a fragility fracture. Although evidence of a definitive causal relationship between bisphosphonate therapy and serious adverse events such as UGIB is lacking, clinicians need to remain cognizant of potential adverse events associated with bisphosphonate use and should routinely ask patients about pre-existing GI disorders and concurrent medication history prior to prescribing these drugs.

## Abbreviations

ADR: Adverse drug reaction; C: Confidence interval; DAD: Discharge Abstract Database; GI: Gastrointestinal; IR: Incidence rate; MSP: Medical services plan; NSAID: Nonsteroidal anti-inflammatory drugs; OR: Odds ratio; PPI: Proton pump inhibitor; UGIB: Upper gastrointestinal bleed.

## Competing interests

None of the authors have any financial or non-financial competing interests to declare as related to the contents of this manuscript.

## Authors’ contributions

Study concept and design: JAK-S, GGC, JH, and DV; acquisition, preparation, analysis, and interpretation of the data: JAK-S and DV; first draft of the manuscript: JAK-S. All authors critically reviewed the manuscript and approved the final version of the manuscript to be published.

## Pre-publication history

The pre-publication history for this paper can be accessed here:

http://www.biomedcentral.com/1471-2318/13/36/prepub

## Supplementary Material

Additional file 1 ICD-9 codes used to identify UGIB cases. Click here for file

Additional file 2 Oral prescription medications included in the analysis. Click here for file
