# Supplementary material for: The association between serious upper gastrointestinal bleeding and incident bisphosphonate use: a population-based nested cohort study
Source: BMC Geriatr. 2013 Apr 20;13:36. doi: 10.1186/1471-2318-13-36 (PMC3653746; doi:10.1186/1471-2318-13-36)
Supplement: Additional file 1 — ICD-9 codes used to identify UGIB cases. [file 1471-2318-13-36-S1.doc]

**Additional file 1**: ICD-9 codes used to identify UGIB cases

| **Diseases of oesophagus** | |
| --- | --- |
| 530.7 | Gastro-oesophageal laceration-haemorrhage syndrome |
| **Gastric ulcer** | |
| 531.0 | Acute with haemorrhage |
| 531.1 | Acute with perforation |
| 531.2 | Acute with haemorrhage and perforation |
| 531.3 | Acute without mention of haemorrhage or perforation |
| 531.4 | Chronic or unspecified with haemorrhage |
| 531.5 | Chronic or unspecified with perforation |
| 531.6 | Chronic or unspecified with haemorrhage and perforation |
| **Duodenal ulcer** | |
| 532.0 | Acute with haemorrhage |
| 532.1 | Acute with perforation |
| 532.2 | Acute with haemorrhage and perforation |
| 532.4 | Chronic or unspecified with haemorrhage |
| 532.5 | Chronic or unspecified with perforation |
| 532.6 | Chronic or unspecified with haemorrhage and perforation |
| **Peptic ulcer** | |
| 533.0 | Acute with haemorrhage |
| 533.1 | Acute with perforation |
| 533.2 | Acute with haemorrhage and perforation |
| 533.4 | Chronic or unspecified with haemorrhage |
| 533.5 | Chronic or unspecified with perforation |
| 533.6 | Chronic or unspecified with haemorrhage and perforation |
| **Gastrojejunal ulcer** | |
| 534.0 | Acute with haemorrhage |
| 534.1 | Acute with perforation |
| 534.2 | Acute with haemorrhage and perforation |
| 534.4 | Chronic or unspecified with haemorrhage |
| 534.5 | Chronic or unspecified with perforation |
| 534.6 | Chronic or unspecified with haemorrhage and perforation |
| **Other** | |
| 578 | Gastrointestinal haemorrhage |
| 578.0 | Haematemesis |
| 578.1 | Melaena |
| 578.9 | Haemorrhage of gastrointestinal tract, unspecified |
| **UGIB** = upper gastrointestinal bleed. | |
