# Supplementary material for: The association between serious upper gastrointestinal bleeding and incident bisphosphonate use: a population-based nested cohort study
Source: BMC Geriatr. 2013 Apr 20;13:36. doi: 10.1186/1471-2318-13-36 (PMC3653746; doi:10.1186/1471-2318-13-36)
Supplement: Additional file 2 — Oral prescription medications included in the analysis. [file 1471-2318-13-36-S2.doc]

**Additional file 2**: Oral prescription medications included in the analysis

| **Non-steroidal anti-inflammatory drugs** |
| --- |
| ketorolac (Toradol) |
| indomethacin (Indocid) |
| naproxen (Naprosyn) |
| celecoxib (Celebrex) |
| diclofenac (Voltaren) |
| rofecoxib (Vioxx) |
| **Antiplatelet / anticoagulant drugs** |
| ASA |
| warfarin (Coumadin) |
| clopidogrel (Plavix) |
| dipyridamole (Aggrenox) |
| ticlopidine |
| **Proton pump inhibitors** |
| omeprazole (Losec) |
| esomeprazole (Nexium) |
| lansoprazole (Prevacid) |
| pantoprazole (Pantoloc) |
| rabeprazole (Pariet) |
